# Atlanta Society of Medicine

**Published:** 1895-11

**Authors:** W. L. Champion

**Affiliations:** Secretary; Atlanta, Ga.


### *SOCIETY REPORTS.*

---

### ATLANTA SOCIETY OF MEDICINE.

ATLANTA, GA., October 15, 1895.

The meeting was called to order by the President, DR. C. D. HURT.

DR. T. D. LOVE read a paper on "La Grippe." He stated that as the disease covered so much territory he would give it only in outline, and would leave the details to be given by the gentlemen appointed to discuss it.

DRS. DUNCAN, HARDON, HAGAN and CREIGHTON had been appointed by the President to discuss the subject, but in the absence of Drs. Hagan and Creighton, it was discussed only by Drs. Duncan and Hardon.

DR. DUNCAN complimented Dr. Love upon his excellent paper, and in discussing the subject stated as follows: "As regards the etiology of the disease, I do not think this is known, and I do not believe in the theory that it is bacteria. I think that it is some condition of the atmosphere which affects the electrical condition of the body. You may take twelve persons and expose them all to the disease, and perhaps only one or two will be attacked by the disease. These will have it probably on account of the condition of the system, and the others will escape. I do not think that it is a germ disease, or that the finding of the bacilli in the blood or other parts of the body during the attack of the disease is any proof that they are connected with the disease. Bacteria are to be found in water, and, also, you can find them in the body whether the person is sick or not. In regard to treatment of the disease, for the non-complicated cases, which, as Dr. Love states, we have the most to deal with, I usually give the following prescription:

℞ Pulv. capsici................................................................ gr. i.
Leptandrin.................................................................. gr. ss.
Phenacetine................................................................. gr. ii.
Pulv. camphor .............................................................. gr. ss.
Sulph. quinine.............................................................. gr. ii.
M. Ft. capsules No. 1. Sig. One every two or three hours.

Something to act upon the liver. Dr. Love states that he gives calomel for this purpose. This does not act upon the liver, but I give neutral salts, which will. When you give calomel and have a dark stool, this is no proof that it acts upon the liver. If the fever is high I give two drops of tincture of veratrum, and in less than six hours the patient will be comfortable without the use of opiates. I do not think you will have any trouble with the heart, even with two and a half grains of phenacetine. However, it is not necessary to give large doses of the coal tar derivatives. Also, I have given a grain or half grain of camphor in the capsules. In regard to complications of the disease, especially of pneumonia, I would state that this is a very serious trouble. In such cases some benefit will be derived from counter-irritation. I would recommend small doses of deodorized tincture of opium. In the regular prescription you can give two and a half grains of Dover's powders, one grain of capsicum, and two and a half grains of quinine, leaving out the coal tar derivative if possible. Some have been fond of using Dr. Miller's remedy, which is the juice of one or two lemons and a good strong drink of rye whisky and going to bed and sweating it out. I have never tried this, but those I have tried have given satisfaction.

Again, we have the sequela of the grippe to deal with as much as the grippe itself. Some months after the attack of the grippe, I have been called to patients to prescribe for vertigo. The patient will be unable to walk across the floor without staggering, and it takes a long time to get over this. I think that often at the first attack of la grippe the electrical machine gets out of order to such an extent that the person never fully recovers. I do not think it is best to purge the bowels too much, but it is good to tone up with quinine, strychnine, and some brandy. However, the prescription mentioned previously will fully answer in many cases of very severe attacks of la grippe, as it has always given satisfactory results.

In discussing the disease from a gynecologist's standpoint, Dr. HARDON stated: "It does not fall to the lot of the gynecologist to treat many cases of la grippe, as these go mostly to the general practitioner. When I do have a case, it is where the case occurs in the course of treatment in the line of my work, or else it is a case in which is described a past attack of the disease. I will mention it only in relation to the pelvic diseases. I will not state as to whether it is due to bacilli. I do not find that the attack of la grippe occurring during the course of a disease is modified by any such complications. That is to say, when a patient has la grippe while under the course of one disease, the course of treatment is the same as if patient were well. In treating these cases of la grippe I treat them the same as if the patient came to me without any pelvic trouble. The treatment is to control the fever and keep up the strength. Perhaps, in the majority of cases, the patient will recover whether treated or not. I think the success of the treatment lies more in contributing to the comfort of the patient, except in cases where it has run to sequela, as in pneumonia. Like Dr. Duncan, I give phenacetine, giving a capsule of three grains, one-half grain of camphor, and one-twelfth of a grain of powdered ipecac and opium. This controls the nervous condition of the patient; also tends to make the patient sleep. I give the camphor for the purpose of controlling the nervous disturbances. The ipecac and opium, of which the patient will get about half a grain in twenty-four hours, is given for the skin. The capsule is to be given every two hours. In giving this prescription the patient is controlled and made to rest. This is the most satisfactory treatment for the general disease. The essential treatment of the disease is the giving of this capsule every two hours. During the progress of la grippe I do not find it necessary to give any additional treatment for the pelvic organs, as the treatment previously given for them is suitable. Now, as to what effect la grippe has on the pelvic diseases, in the majority of cases my experience has been that it has no effect. But there are some exceptions. I have found cases where the patients were prone to the enlargement of the pelvic organs that this is apparently aggravated by the appearance of la grippe. Patients who have been treated for pelvic diseases, and have recovered, will have a reappearance of this disease with the occurrence of la grippe, but I have been unable to recall a disease which resulted from la grippe. Diseases which are affected by la grippe affect only the mucous membrane. Last summer I had a patient who suffered with enlargement of the womb, and who told me the symptoms originated soon after the grippe; but some four or five months previous to the attack of the grippe she had given birth to a child, and this birth had left her with a severe laceration of the peritoneum. I think that in many cases, as in this instance, the occurrence of many symptoms may be a coincidence, rather than a case of cause and effect. In cases which seem to have set up anew by la grippe, I know of no treatment other than that which would be given for the original case. If they do not yield to the treatment they go on with the same results. There is no modification of the course of the disease or the effect of the treatment to it. So that, really, it narrows the subject down to a small space in relation to the gynecologist. All that we can say is, that in some rare cases a persistent disease that had been apparently cured is set up anew by the appearance of la grippe."

DR. KENDRICK stated: "I have nothing new to add to what has already been stated. We have the definitions of the disease as described by Dr. Love, and I think they were well defined. One class affects the respiratory organs, and another affects the digestive, and then affects the nervous system. I have had several cases within the last week, but cannot say whether they were genuine types. I was called to see a young lady Saturday who was suffering with pains in the back, temperature 103 and 104, and all the evidences of la grippe. I used the treatment as mentioned by Drs. Hardin and Duncan, and she recovered at once. In regard to causes of this trouble, I presume none of us can state the causes, but I am of the opinion that it is a germ disease, but cannot back this with any knowledge of it; but it seems that this is the most plausible theory. There is no question in my mind that it is due to bacilli. It makes its appearance along the avenues of travel. It crops out in large cities, and seems to be of germ origin, and that we inhale it. It is reasonable that la grippe appears from inhalation. I have been in an epidemic, treating several hundred cases, and have no recollection of a complication of pneumonia. In treating cases, we can make the patient comfortable and help him in this way. If I knew that we would have an epidemic of la grippe within a few weeks, the epidemic itself would not trouble me so much as the after effects of it. I have two or three cases at present which seem to be nothing more than the after effects of la grippe. I find that the class of people who are susceptible to tubercular diseases and various other affections did not have anything of this kind until having an attack of la grippe, and months and years afterwards we find these symptoms of la grippe cropping out. I find patients affected with rheumatism and other troubles which can be traced back to la grippe. It affects persons of all ages."

DR. KIME stated: "I do not know exactly the causes of la grippe, but from its history I think it is due to germs and germ development. I can speak from a practical experience, and know that a person can suffer as much pain in a few hours as from anything I know of. The treatment of the disease as previously outlined is to give relief to the patient. In my case I would do most anything to get relief. I have found that the treatment as outlined to-night has been very beneficial. I have used one grain of Dover's powders and one grain of phenacetine, not more than two. This acts well. It seems to me that the complications will be in accordance with the conditon of the patient previous to the attack. In other words, if the patient has no trouble previously, he will recover without any difficulty, but if he has some previous affection will expect this trouble to be aggravated by an attack of la grippe. All have seen cases of tuberculosis which were brought about by an attack of la grippe, and we have seen how it expends its force on organs that have been previously diseased. I cannot say that an attack of la grippe originates a disease."

DR. HURT stated as follows: "In regard to the germ theory, I think that the disposition of la grippe is to take hold of the entire system, affecting the blood, nerves, etc. Where a man or woman is regarded as being in perfect health, they are hardly subject to the complications. I believe that la grippe lowers the vital force and energies of the system, and I think that on this account the dangers of sequela are so frequent. In the month of January, 1891, I had several hundred cases of la grippe. Would go to a house and find from four to ten cases in the house. My prescription was four grains of phenacetine, and from one-eighth to one-fourth of a grain of codeine and capsicum, and when I did not use this I used camphor. This was given from three to four hours, and would soon quiet the patient, and in twenty-four hours the condition would be very much relieved and often had no occasion to repeat the prescription. There were others who had an impaired condition of the lungs and the nervous system, and these patients gave more trouble and required more attention. I want to call attention to one thing that I noticed in these cases. During the month of January I was called to these several hundred cases. In the month of February they were quiet, but in March there was a general noticeable disturbance among these patients. They seemed to have an apparent relapse. As to whether the weather affected them, I cannot say, as I did not take this into consideration. I think that young people and those who have not reached the age of forty recover more speedily than those beyond that age. I have been almost inclined to the opinion that beyond this age a person never regains the powers of the system lost in an attack of la grippe. They may, but it will take a long time. On the first day of January, 1892, I was taken with a very hard chill about 10 o'clock in the morning. I was out driving at the time and went home at once and went to bed, and in twenty minutes was delirious and remained so for five days. The physician told me I had bronchitis and had come near dying, but as a result of this attack I had rheumatism in the back and suffered intense pain. In the summer I went to a resort but this did not seem to help it. Soon after I moved to Atlanta, and then had a second attack, and this was followed with catarrhal jaundice. I am confident that I have never been myself since January, 1892."

DR. LOVE stated: "I cannot add much to what has already been said, but a few points have been mentioned that I would like to refer to. Dr. Duncan stated, and I agree with him, that we do not know what the disease is, but we have to take the statement of responsible authors who have examined the disease from a scientific standpoint. I have looked up the disease thoroughly, and the majority of the writers give the origin as due to bacilli. Some simple and complicated cases have been examined, and cases complicated with bronchitis and pneumonia, etc., and there have been found germs sufficient to produce pneumonic condition in rats. And also state that parties living in the suburbs of infected districts, that those who come into the city and mingle with the infected sections, go back home and are the first to have the disease and then it spreads to the other inhabitants of the suburb. They claim by this that it is an infectious disease. Others go on to state that there is a miasmatic condition and acts through the atmosphere and is propagated that way. In cases of la grippe, where there is a predisposition to lung trouble, the lungs are affected, and so in other diseases. My experience has been that the treatment of it is, that those patients who are first attacked with simple la grippe, if they call in a physician, and he examines the case and makes proper prescription and sends the patient to bed until vitality is restored, the patient will not have any complications. Other cases that are not treated by a physician have complications that pass off in four or five days and leave the patient in a prostrated condition. One way that the complications are acquired is that the patient gets up too soon and has a relapse. In 1890 I had an attack of la grippe that was of the catarrhal form. I had high fever and profuse vomiting, which could be controlled only by injections of morphine. I had an abscess in my right ear. I have considered that I have not had a well day since then. I have a cough and catarrhal condition of the stomach. Organs are all upset, and I have lived five years hoping to outgrow the trouble but have never succeeded in doing so. As to treatment, we all have our different lines of treatment. My favorite prescription is to give calomel and soda, and follow this with a prescription of half a grain of caffein, given every three hours. Watch the patient closely, and if the temperature is not reduced, give two grains of phenacetine. Never give large doses of the coal-tar derivatives, as small doses give best results, and give more frequently than large doses. Use all the stimulants that the patient will bear. This is for simple cases. In complicated cases you have to treat accordingly. In the treatment of pneumonia I have had the most favorable results from large doses of quinine, with stimulants, and sufficient amount of anodynes to control the patient and keep comfortable. As to catarrhal condition of the stomach, you have to treat that with the preparations that will relieve that condition. I have found beneficial results with the administration of bismuth and lactopeptin, but cannot define any definite line of treatment that will cover all cases. You have to take each case separately and distinctly. One particular point is that we fail to keep the patients confined after they are able to get up and about. A person who is confined with a disease and gets up, will take advantage and go out too soon, and in this lies the danger of la grippe; too early exposure and want of treatment after the disease has apparently disappeared. I usually give a supportive prescription after I have dismissed them, and keep them on this from four to six weeks."

W. L. Champion, M.D., Secretary.

